# Capability Study of Ti, Cr, W, Ta and Pt as Seed Layers for Electrodeposited Platinum Films on *γ*-Al_2_O_3_ for High Temperature and Harsh Environment Applications

**DOI:** 10.3390/ma10010054

**Published:** 2017-01-11

**Authors:** Marietta Seifert, Erik Brachmann, Gayatri K. Rane, Siegfried B. Menzel, Thomas Gemming

**Affiliations:** SAWLab Saxony, IFW Dresden, P.O. Box 270116, 01171 Dresden, Germany; e.brachmann@ifw-dresden.de (E.B.); g.k.rane@ifw-dresden.de (G.K.R.); s.menzel@ifw-dresden.de (S.B.M.); t.gemming@ifw-dresden.de (T.G.)

**Keywords:** surface acoustic waves, high temperature sensors, planar Pt antenna, electrodeposition, Al_2_O_3_

## Abstract

High temperature surface acoustic wave sensors based on radio frequency identification technology require adequate antennas of high efficiency and thermal stability for the signal transmission. Platinum is well known and frequently used as a material of choice for high temperature and harsh environment applications because of the high melting point and its chemical stability. Therefore, one way to realize high temperature stable antennas is the combination of a Pt metallization on an Al${}_{2}$O${}_{3}$ substrate. As a cost-effective technique, the Pt film is deposited via electrochemical deposition. For this growth procedure, a pre-deposited metallization on the Al${}_{2}$O${}_{3}$ layer is required. This paper analyzes the influence of various seed layers (Ta, Ti, W, Cr, Pt) on the morphology, stability and electrical properties of the electrochemically-grown Pt thick film after heat treatments up to 1000 ${}^{\circ }$C in air. We find an oxidation of all adhesion layers except for Pt, for which the best electrical properties were measured. Although significant areas of the films delaminate from the substrate, individual anchor structures retain a stable connection between the Pt layer and the rough Al${}_{2}$O${}_{3}$ substrate.

## 1. Introduction

In recent years, there has been a growing development of Pt-based sensors [1,2,3], such as temperature sensors, chemical sensors [4] and biological sensors [5,6], because of the outstanding high temperature properties and corrosive resistance of this material. One way to realize such sensors is the surface acoustic wave (SAW) technology, which has been used since more than 30 years for high precision frequency filters [7]. The application of SAW devices for high temperature sensing also combined with radio frequency identification (RFID) has been demonstrated several times [1,8,9,10]. Such a passive sensor based on SAW technology is composed of a piezoelectric substrate with interdigital transducer electrodes connected to an antenna consisting of a structured metallization on an insulating substrate. All components have to be high temperature stable. In this paper, we focus on the antenna system.

A combination of physical vapor deposition (PVD) processes (especially e-beam deposition) with the lift-off procedure is the standard structuring technique for biological, electrochemical and interdigital transducers [11]. This standard procedure can also be applied for the fabrication of high temperature antennas on rigid high temperature-stable ceramic substrates, such as *γ*-Al${}_{2}$O${}_{3}$. If a noble metal is deposited on an oxidic substrate, adhesion problems will generally occur because of its bad sticking behavior, leading to agglomeration and delamination processes during a heat treatment. A general overview of delamination effects is given, e.g., by Khanna [12]. Classical adhesion layers like titanium that increase the stability of platinum films were described in [13,14]. However, at high temperature and harsh environmental conditions, titanium interacts with platinum, leading to migration and oxidation effects [15,16]. For polished *γ*-Al${}_{2}$O${}_{3}$-substrates and sputtered platinum, the TiO${}_{x}$ adhesion layer has an effect on the texture and the stability of the Pt-film [17]. Interdiffusion between Pt and Ti films under oxygen atmosphere is shown in [18,19,20]. Tisone and Drobek found diffusion factors of Ti in Pt 10${}^{5}$-times higher along grain boundaries compared to the bulk value [21].

Zr and Ti have been used as adhesion layers for platinum metallization, e.g., on smooth sapphire substrates (root mean square roughness $R_{q}=1.3\pm 0.2$ nm) [14], polycrystalline alumina oxide (arithmetic average roughness $R_{a}=35$ nm) [4,22] or commercial Si ($R_{q}<1$ nm) [23].

The above-mentioned results refer to PVD, e.g., sputtered or e-beam deposited, films. An alternative to PVD is the electrochemical deposition or electroplating, which reduces costs compared to standard PVD processes. The biggest benefit compared to PVD processes where a huge amount of material condenses amongst others on the vacuum chamber walls is that the deposition of material only occurs on electrically-conducting surfaces that are in contact with the electrolyte. Especially for expensive materials like platinum or other noble metals, electroplating can be a cost-effective alternative. Therefore, this paper reports on a combination of standard PVD deposition processes for thin seed layers with the low cost electrodeposition technique for the growth of the thick working metallization. The focus is to study the electrochemically-deposited Pt films with the application of various seed (Ti, W, Ta, Cr, Pt) layers. These bilayers are characterized concerning their high temperature stability for application as an antenna system in high temperature SAW sensor devices.

## 2. Experimental Section

All layers were deposited on rectangular *γ*-Al${}_{2}$O${}_{3}$-substrates (Rubalit 708S, CeramTec GmbH, Marktredwitz, Germany) with a thickness of 0.6 mm and a surface roughness with maximum values of $R_{a}=600$ nm according to the manufacturer. After cutting the ceramic plates to samples with dimension 24 mm by 15 mm the pieces were cleaned in aqua regia at 80 ${}^{\circ }$C for 1 h and then boiled two times in deionized water for 1 h. Afterwards the samples were baked in a high temperature oven at 1200 ${}^{\circ }$C under ambient atmosphere to reduce residual organic contamination by forming gaseous organics. The combination of the wet chemical cleaning process and the high temperature treatment should remove metallic and organic contaminants from the samples surface. The ceramics were removed from the oven at a temperature above 120 ${}^{\circ }$C and transferred to the device for the deposition of the seed layers. There, a final temperature treatment took place at 500 ${}^{\circ }$C for 2 h under high vacuum (HV) atmosphere. The deposition device (CREAMET 350-CL 6, CREAVAC—Creative Vakuumbeschichtung GmbH, Dresden, Germany) with a base pressure below $2\times 10^{-4}$ Pa is evacuated by a combination of Roots pumps (ACP 28, Adixen Pfeiffer Vacuum GmbH, Asslar, Germany) and magnetically levitated turbopumps (TURBOVAC MAG W 600 iP, Oerlikon Leybold Vacuum GmbH, Dresden, Germany).

The nominal necessary film thickness to achieve closed seed layers on polished substrates is about 10 nm. In our case, the substrate roughness is in the same range as the targeted film thickness, hence a seed film thickness of about 100 nm was chosen. The seed layers also convey the adhesion between the Pt metallization and the substrate. The Ti, W, Ta and Cr seed layers that are investigated in this study are sputter deposited at room temperature at a pressure of 0.18 Pa by adding 30 sccm Ar sputter gas with a purity of 99.999. The 100 nm platinum seed layer is electron beam evaporated (EVM-6, FERROTEC GmbH, Untersingen, Germany) in a different chamber of the device at a base pressure of $2\times 10^{-5}$ Pa. The e-beam deposition was chosen for Pt since less of this highly expensive material is required for this deposition technique compared to the sputtering. Deposition rates of the PVD-films are summarized in Table 1. Finally, a thick Pt layer was electrodeposited. This galvanic platinum film is indicated as Pt${}^{*}$ in the following.

The seed layer-substrate compounds were stored under high vacuum in the PVD-device until the galvanic deposition process was carried out to prevent oxidation of the materials. To contact the seed layer electrically with a power supply, alligator clamps were directly connected to the metallization. The counter electrode was a circularly curved Pt sheet. The deposition took place in a 100 mL beaker filled with 50 mL commercial platinum electrolyte (Platinbad JE18, C. Jentner GmbH, Pforzheim, Germany), which has a platinum content of 2 g/L solved in a 10% sulfuric acid with a pH value of 1.5. The solution was convected by a magnetic stirrer at 1200 min${}^{-1}$ on a hotplate with a bath temperature of 30±1 ${}^{\circ }$C. The galvanostatic deposition process was controlled by a constant current source (6220-DC current source, KEITHLEY-TEKTRONIX Inc., Beaverton, OR, USA) at 30 mA resulting in a current density of 10 mA/cm${}^{2}$. The process time was about 750 s. The Pt${}^{*}$ films were prepared with a thickness between 1 and 2 μm.

After preparation, the samples were heated in air in an oven (rapid high temperature furnace, Kanthal Super HT, SANDVIK AB, Hallstahammar, Sweden). The heating rate was 10 K/min, the dwell time 2 h and thereafter the samples were cooled down within the oven until the transfer temperature of approximately 120 ${}^{\circ }$C was reached. The annealing temperatures were 400, 600, 800 and 1000 ${}^{\circ }$C.

The phase formation of the Pt films has been investigated by X-ray diffraction (XRD, Philips X’Pert PW3040/00, Co-Ka, PANalytical, Almelo, The Netherlands) in Bragg Brentano geometry.

Measurements of the electrical resistance were carried out by using the van der Pauw measurement method for thin films on planar substrates [24]. Several measurements were performed with constant current of 5 and 10 mA and changing the current direction for each measurement. The current was measured by a nanovoltmeter (2182A—Nanovoltmeter, KEITHLEY-TEKTRONIX, Inc., Beaverton, OR, USA).

Cross-sections of the film-substrate compounds were prepared by the focused ion beam technique (FIB, Zeiss 1540 XB CrossBeam) and top down images were acquired by using scanning electron microscopy (SEM, Zeiss Ultra Plus) both from Carl Zeiss Microscopy GmbH, Jena, Germany.

Transmission electron microscopy (TEM, FEI Technai F30, FEI, Hillsboro, OR, USA) was applied to image the grain morphology. The local chemical composition was analyzed in the same instrument with energy dispersive X-ray spectroscopy (EDX, EDAX, Mahwah, NJ, USA). Additional to conventional TEM imaging bright field as well as high-angle annular dark field scanning modes (BF-/HAADF-STEM) were applied either to image the grain orientation or the chemical contrast (the imaging contrast used in each image is denoted in each case).

## 3. Results

### 3.1. Phase Formation

**Figure 1:**
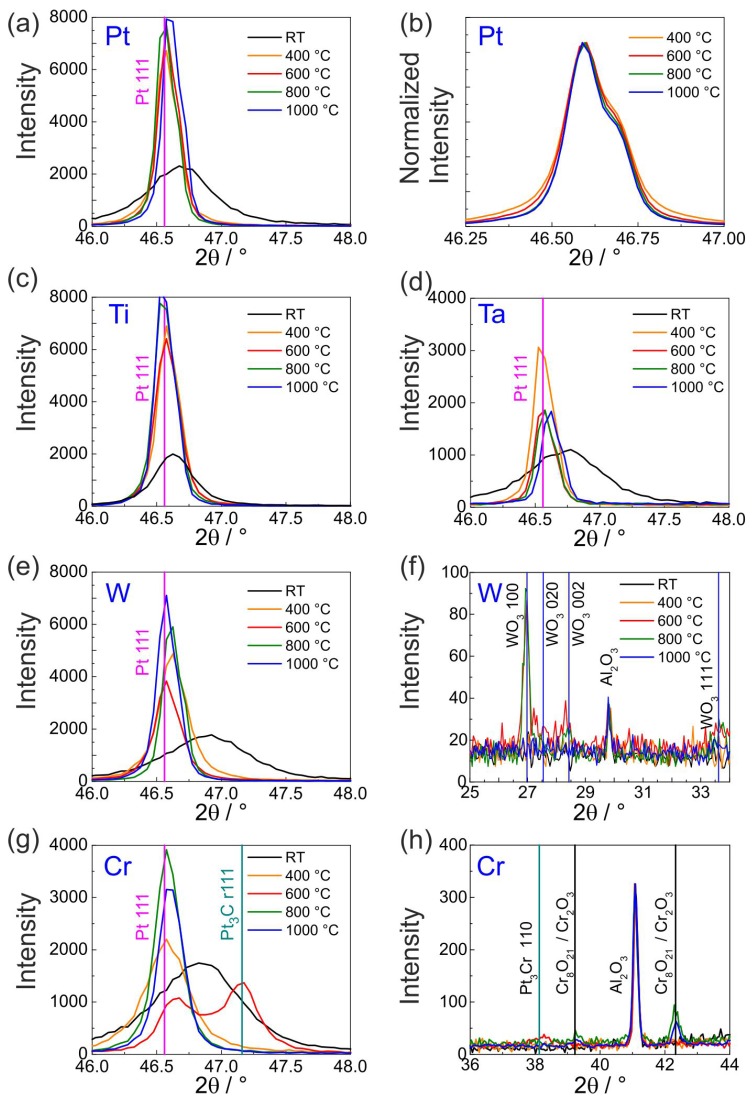
XRD diagrams of the Pt${}^{*}$ films on various seed layers in the as-deposited state (RT) and after annealing at 400, 600, 800 or 1000 ${}^{\circ }$C in air. (**a**) Pt; (**b**) Pt with normalized intensities for a better comparability of the peak width; (**c**) Ti; (**d**) Ta; (**e**,**f**) W showing the Pt and WO${}_{3}$ reflexes; (**g**,**h**) Cr showing the Pt, Pt${}_{3}$Cr and Cr oxide reflexes.

Figure 1 shows the results of the XRD measurements of the Pt${}^{*}$ films with Ti, W, Ta, Cr or Pt seed layer in the as-deposited state (denoted as RT) and after the annealing steps.

In all the films in the as-deposited state broad peaks can be seen, which are caused by the small grain size (see also cross-section images in Figures 3, 5, 7, 9 and 12 for the different material systems). All the peaks are shifted to higher angles as compared to the standard position (for stress-free bulk material) suggesting that the films are under tensile stresses. Only in the case of Pt${}^{*}$ on Ti this effect is less pronounced. As the samples are annealed, the peaks are clearly shifted to the Pt standard position and the peak intensity increases along with narrowing of the peaks. This can be attributed to grain growth as is also confirmed from the FIB-cut cross-section view of the films (see Section 3.2). The narrowing of the peaks upon annealing can be well appreciated from the exemplary Pt 111 peak (intensity normalized for viewing) shown in the Figure 1b. Above 800 ${}^{\circ }$C, only a very small narrowing can be ascertained.

In all the films, changes in the diffractogram are visible above 600 ${}^{\circ }$C. In case of W and Cr seed layer, clear oxide peaks belonging to WO${}_{3}$ (at 600 and 800 ${}^{\circ }$C, Figure 1f) and Cr${}_{8}$O${}_{21}$ or Cr${}_{2}$O${}_{3}$ (at 800 and 1000 ${}^{\circ }$C, Figure 1h) are indicated. However, in Ti and Ta (Figure 1c,d) the phase of the extra peaks (positions not shown here) could not be affirmatively established. In the Pt/Cr film, Pt${}_{3}$Cr peaks were observed at 600 ${}^{\circ }$C that disappear at higher temperatures (Figure 1g).

**Figure 2:**
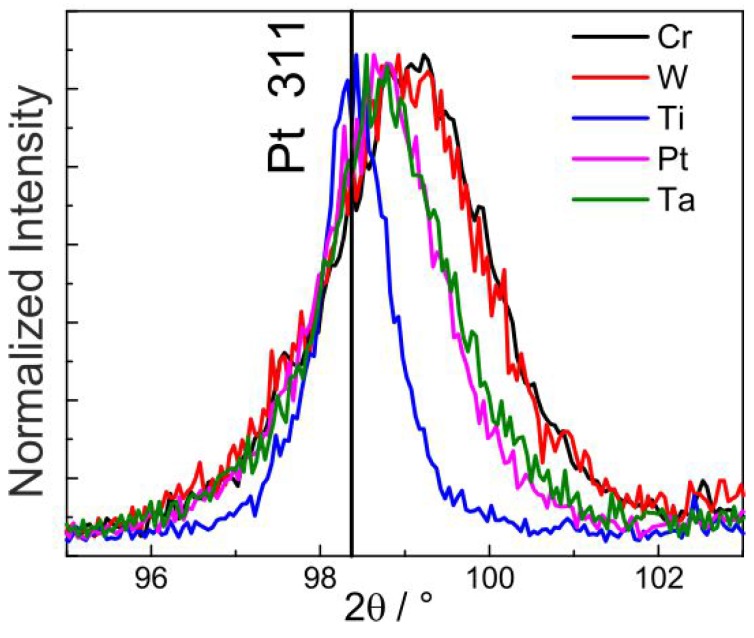
Comparison of the Pt peak width for the Pt${}^{*}$ films with the various seed layers in the as-deposited state.

Figure 2 shows the normalized Pt 311 peak for all the as-deposited samples. The quantity of peak shift to higher angles as well as the increase in the peak broadening follow the order Ti < Pt ≈ Ta < Cr ≈ W. This order corresponds to the increasing tensile stress and decreasing grain size in the as-deposited Pt films. Thus, Pt films in the as-deposited state on Cr and W had the smallest grain size. This is also re-confirmed from the cross-section images.

### 3.2. Film Morphology

#### 3.2.1. Ti Seed Layer

**Figure 3:**
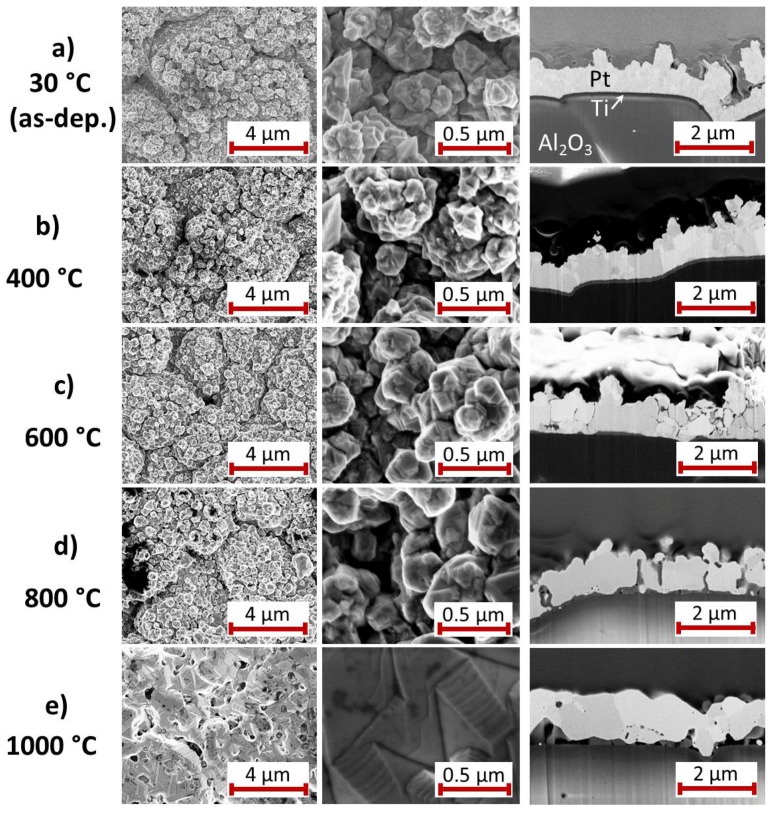
SEM images (InLens, 5 kV, 3 mm working distance) with two different magnifications of the surface of the electrodeposited Pt${}^{*}$ film on Ti seed layer (**a**) after deposition and after annealing for 2 h in air at (**b**) 400; (**c**) 600; (**d**) 800 and (**e**) 1000 ${}^{\circ }$C. On the right hand side a SEM image (InLens, 3 kV, 5 mm working distance) of the film cross-section is presented.

For the Pt${}^{*}$ film with Ti seed layer, Figure 3 summarizes the results of the SEM study of the surface and the cross-section images for the as-deposited state and after annealing at various temperatures. The images of the as-deposited samples (Figure 3a) show a Pt${}^{*}$ film with grains of the size between 10 and 100 nm and a clear interface to the Ti seed layer. A peculiarity of this film compared to the others is the growth of Pt whisker-like structures. These structures are formed if the electrodeposition is carried out in an electrolyte with low Pt content. This effect has been avoided for the subsequent depositions, as can be seen in the SEM images in the following sections.

The heat treatment at 400 ${}^{\circ }$C leads to a clear grain growth within the Pt${}^{*}$ film whereas there are hardly any changes at the film surface (Figure 3b). The Ti film is still visible as a separate layer.

The high magnification SEM image of the 600 ${}^{\circ }$C annealed sample (Figure 3c) shows that grains have further increased in size. The cross-section image demonstrates that the contrast of the grain boundaries has become significantly stronger. The Ti seed layer remains visible only below the extended grain in the center of the image. It has to be assumed that this annealing temperature is sufficient to activate the diffusion of Ti atoms along the Pt${}^{*}$ grain boundaries, as mentioned in the introduction (see Section 1). In contrast to this, after annealing at 800 ${}^{\circ }$C (Figure 3d) the grain boundaries are hardly visible. Instead, a few narrow darker grains appear, which are extended nearly across the film thickness and, additionally, are present at some points between the Pt${}^{*}$ film and the substrate.

The most considerable changes are visible after annealing at 1000 ${}^{\circ }$C (Figure 3e). The surface morphology has changed completely. The surface is much smoother, extended facets have formed and the whisker-like structures disappeared completely. The cross-section shows grains with μm size. However, the film has delaminated locally from the Al${}_{2}$O${}_{3}$ substrate. As for the 800 ${}^{\circ }$C annealed sample, also dark grains are visible between the substrate and the film and locally within the Pt${}^{*}$ grains.

**Figure 4:**
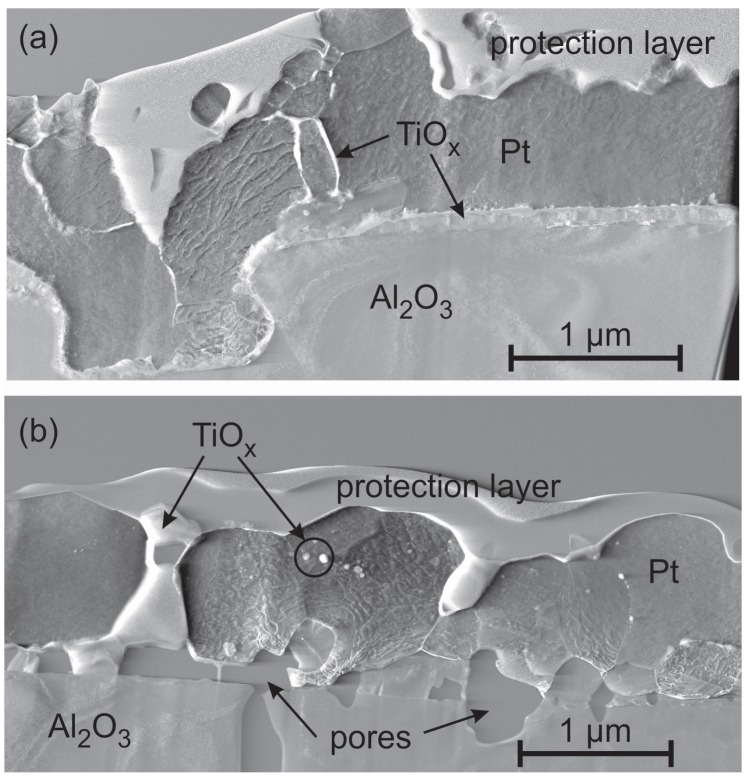
STEM image (grain orientation contrast) of the Pt${}^{*}$ film with Ti seed layer after annealing (**a**) at 600 ${}^{\circ }$C and (**b**) at 1000 ${}^{\circ }$C in air.

To get more information about the film structure after the annealing process TEM studies have been performed for the 600 ${}^{\circ }$C and 1000 ${}^{\circ }$C annealed samples (Figure 4). The protection layer, which is labeled in Figure 4 and all further TEM images, is deposited within the FIB device prior to the cutting of the TEM lamella to protect the surface of the film during the preparation procedure.

For the 600 ${}^{\circ }$C sample (Figure 4a) the EDX measurements prove that Ti is diffusing along the grain boundaries towards the sample surface. Already at this temperature Ti is oxidized. However, most of the TiO${}_{x}$ is still located at the interface between the Al${}_{2}$O${}_{3}$ substrate and the Pt film.

The sample annealed at 1000 ${}^{\circ }$C (Figure 4b) shows extended μm size Pt grains and confirms the presence of several 100 nm sized pores between the film and the substrate, which are formed by the outwards diffusion of Ti. At several positions TiO${}_{x}$ grains are observed between the Pt${}^{*}$ film and the substrate and also between the Pt${}^{*}$ grains and they reach up to the film surface. Locally, TiO${}_{x}$ grains with a size in the range of 10–100 nm are embedded in the Pt${}^{*}$. Although a small solubility (1–5 at % of Ti in Pt at 1000 ${}^{\circ }$C, depending on the source) for Ti in Pt is expected from the Pt-Ti phase diagram [25,26], we only find pure Pt grains without Ti. This can be explained by the high affinity of Ti for O so that it is oxidized instead of being solved within the Pt matrix. The Ti-O phase diagram shows that there are many different Ti oxide phases [27]. For us it was not possible to determine which phase or which mixture of phases has formed.

#### 3.2.2. W Seed Layer

**Figure 5:**
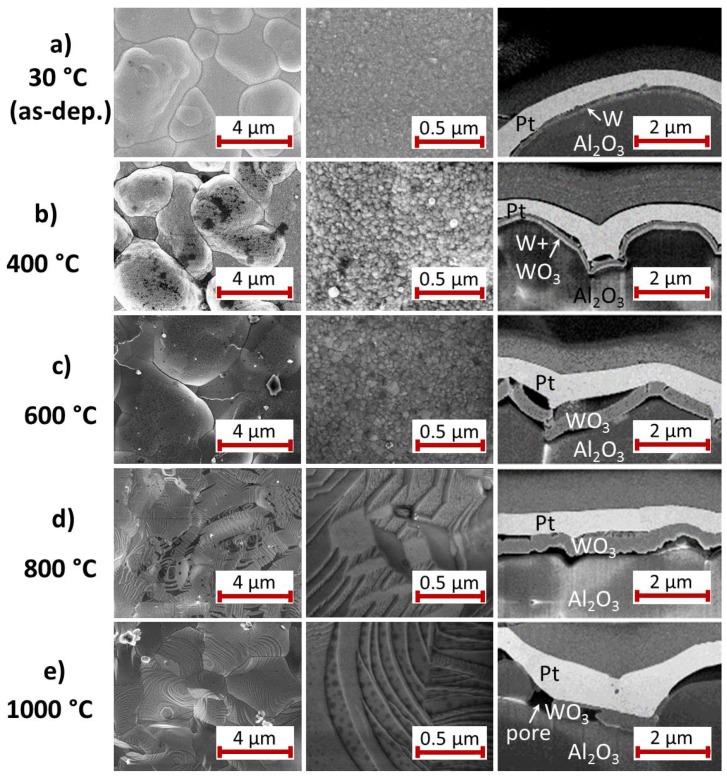
SEM images (InLens, 5 kV, 3 mm working distance) with two different magnifications of the surface of the electrodeposited Pt${}^{*}$ film on W seed layer (**a**) after deposition and after annealing for 2 h in air at (**b**) 400; (**c**) 600; (**d**) 800 and (**e**) 1000 ${}^{\circ }$C. On the right hand side a SEM image (InLens, 3 kV, 5 mm working distance) of the film cross-section is presented.

The behavior of the Pt${}^{*}$ film on W seed layer (Figure 5) during the annealing process is different to that observed on Ti.

Comparable to the Ti sample, after deposition (Figure 5a) the Pt${}^{*}$ film is very fine grained (grains with a few tens of nm size) with a smooth surface and the separate W film is clearly visible. Furthermore, the heat treatment at 400 ${}^{\circ }$C leads to a strong Pt grain growth (Figure 5b). The cross-section image, however, indicates that the Pt${}^{*}$ layer starts to delaminate from the W layer at this temperature. Additionally, the seed layer possesses a two-layer structure with a brighter lower and a darker upper layer. The total thickness of the seed layer has slightly increased.

In contrast to this, after annealing at 600 ${}^{\circ }$C, the seed layer has increased its thickness to approximately three times the thickness of the former W layer (Figure 5c). Metallic tungsten gets oxidized at temperatures above 400 ${}^{\circ }$C [28]. The bilayer structure of the W seed film after the heat treatment at 400 ${}^{\circ }$C can be explained by the initiation of the oxidation whereas at 600 ${}^{\circ }$C, W is oxidized completely. The density of the formed WO${}_{3}$ is much smaller than that of metallic W (6.47 g/cm${}^{3}$ compared to 19.3 g/cm${}^{3}$). In WO${}_{3}$ the volume per W atom is therefore about three times as high as for the W film, leading to the observed increase in film thickness. The origin of the oxygen for the formation of WO${}_{3}$ will be discussed in Section 4.

**Figure 6:**
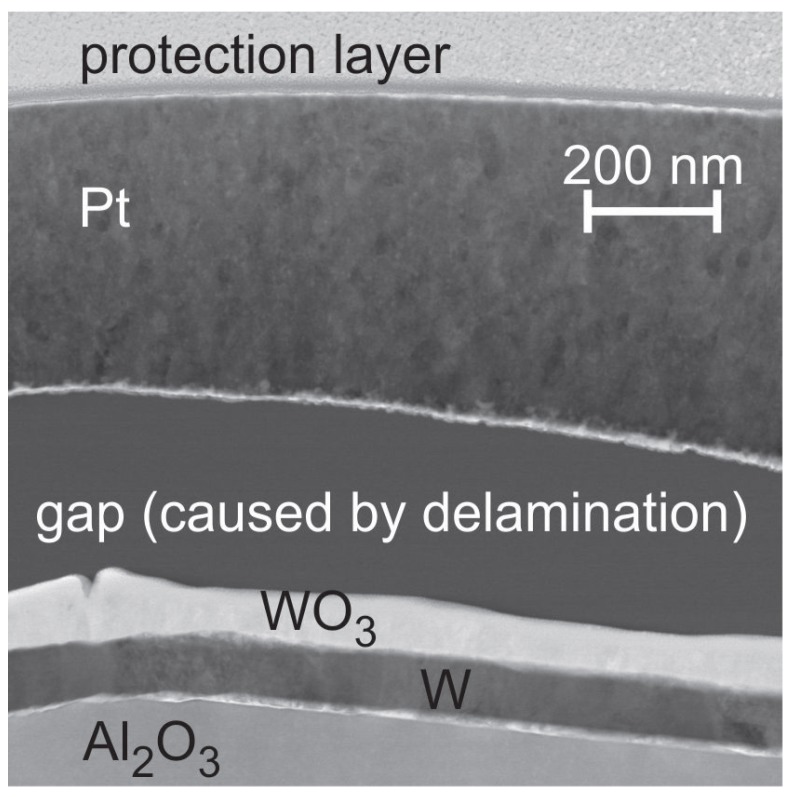
HAADF STEM image (chemical contrast) of the Pt${}^{*}$ film on W seed layer after annealing at 400 ${}^{\circ }$C in air. Due to the high thickness of the TEM lamella the absorption of the electrons in the Pt${}^{*}$ and W layers plays an important role leading to the dark appearance of W and Pt and to the brighter appearance of the WO${}_{3}$ layer and Al${}_{2}$O${}_{3}$ substrate.

To answer the question whether the W seed layer oxidizes from the substrate or the Pt${}^{*}$ film side, a TEM investigation with EDX analysis was carried out for the 400 ${}^{\circ }$C sample. The HAADF STEM image (chemical contrast) in Figure 6 clearly shows the bilayer structure of the seed layer as well as a large gap between the seed layer and the Pt${}^{*}$ film. The EDX analysis reveals that the upper part of the seed layer is composed of WO${}_{3}$, which means that the oxidation occurs from the top side.

After annealing at 600 ${}^{\circ }$C the Pt${}^{*}$ film is strongly delaminated. It appears that its shape does not correlate any more with the huge roughness of the substrate, but that instead this morphology is leveled out. The driving force for this behavior might be the resulting decrease of surface energy. Large grains (μm size) have formed and a contrast indicating smaller grains is only visible where the film is bent outwards. At those positions heating of the sample leads to compressive stress. This can be reduced by the formation of small grains, since a large density of grain boundaries allows reduction of the mechanical stress.

After annealing at 800 ${}^{\circ }$C (Figure 5d) and 1000 ${}^{\circ }$C (Figure 5e) large flat planes are observed at the sample surface and clear facets have formed. The formation of these facets occurs at lower temperatures as compared to the Ti seed layer. Especially after the 1000 ${}^{\circ }$C heat treatment the WO${}_{3}$ layer is not continuous any more but contains large pores, which can be explained with the sublimation of WO${}_{3}$. According to [29] the sublimation of WO${}_{3}$ starts at 750 ${}^{\circ }$C and is significantly stronger above 900 ${}^{\circ }$C.

#### 3.2.3. Ta Seed Layer

**Figure 7:**
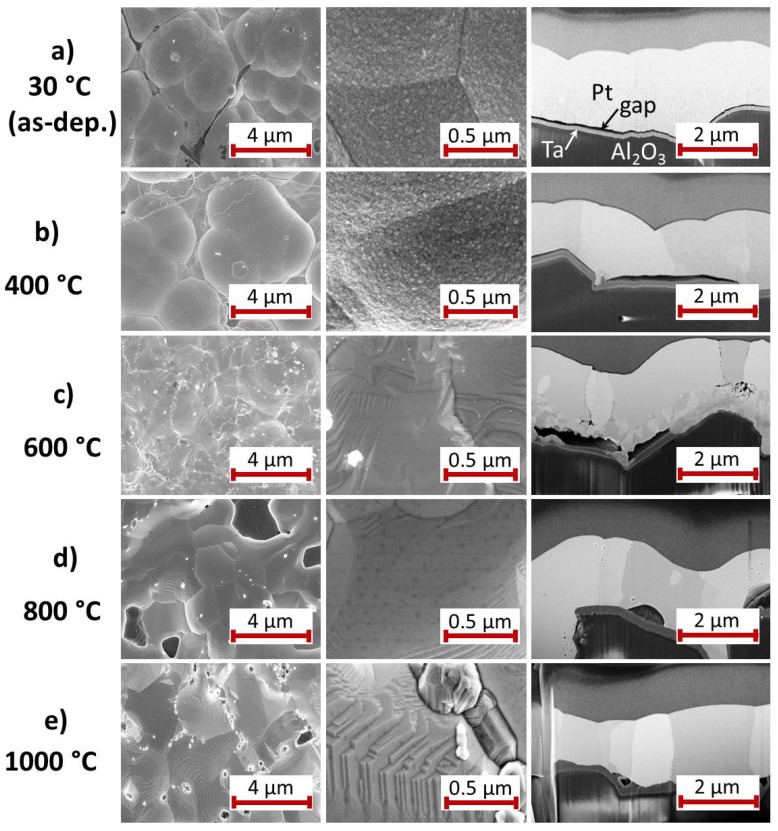
SEM images (InLens, 5 kV, 3 mm working distance) with two different magnifications of the surface of the electrodeposited Pt${}^{*}$ film on Ta seed layer (**a**) after deposition and after annealing for 2 h in air at (**b**) 400; (**c**) 600; (**d**) 800 and (**e**) 1000 ${}^{\circ }$C. On the right hand side a SEM image (InLens, 3 kV, 5 mm working distance) of the film cross-section is presented.

The morphology of the Pt${}^{*}$ film on Ta seed layer is presented in Figure 7. In contrast to the above described films, at RT there is hardly any contact between the Ta seed layer and the Pt${}^{*}$ film (Figure 7a). The heat treatment at 400 ${}^{\circ }$C (Figure 7b) locally enlarges the size of these gaps. Only at individual points there is a contact between the Pt${}^{*}$ and the Ta. Furthermore, the thickness of the Ta layer has increased and comparable to the W seed layer there is a clear contrast between an upper and lower part of the layer. At RT there is a fine grain structure (few tens of nm) in the Pt${}^{*}$ film. After annealing at 400 ${}^{\circ }$C, large grains with μm size have formed.

Annealing the sample at 600 ${}^{\circ }$C leads to a completely different microstructure (Figure 7c). In the upper region, there are large Pt grains. In the lower region there is a zone with grains in the size of 100 nm. In places the separate seed layer is present and above this layer big gaps are visible. On the right hand side of the image the 100 nm sized grain zone is extended to the substrate and no seed layer is identifiable at this position. This indicates a diffusion of Ta atoms in the Pt${}^{*}$ film. In contrast to Ti, Ta has a much higher solubility in Pt. At 600 ${}^{\circ }$C there is a solubility of about 15 at % of Ta in Pt [30], which can explain the formation of the different grain morphology in the lower film region.

**Figure 8:**
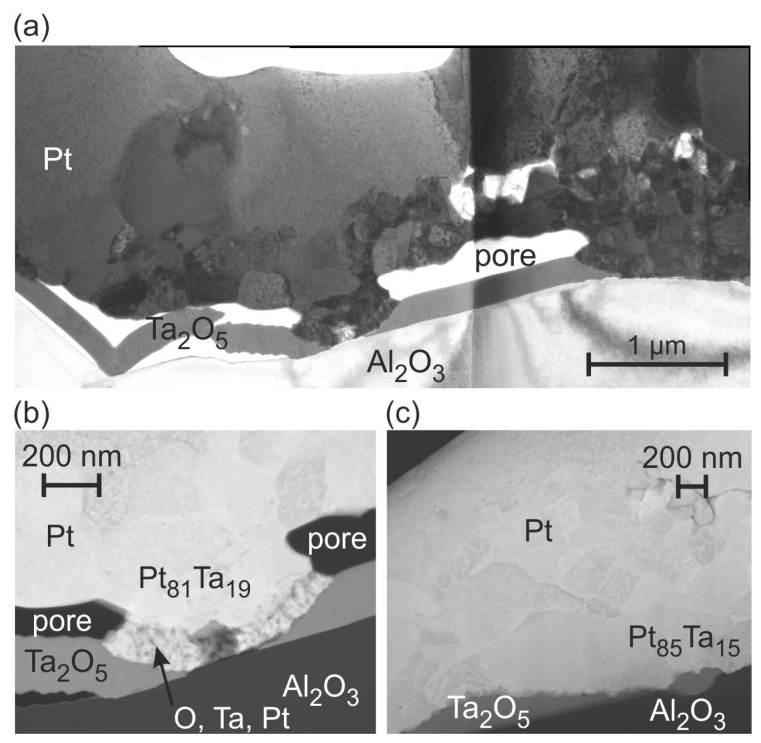
(**a**) TEM image of the Pt${}^{*}$ film on Ta seed layer after annealing at 600 ${}^{\circ }$C in air; (**b**,**c**) HAADF STEM images (chemical contrast) with results of the EDX analysis.

The TEM/STEM images and EDX measurements presented in Figure 8 confirm the dissolution of Ta in the Pt${}^{*}$ grains and the oxidation of Ta. The overview TEM image in Figure 8a illustrates the smaller grains in the lower region of the Pt${}^{*}$ film, whereas in the upper region large grains are present. The EDX results (Figure 8b,c) confirm the diffusion of Ta into the Pt${}^{*}$ layer. Of course, this interdiffusion only takes place at positions with a direct contact between the Pt${}^{*}$ and the Ta film. At regions without a contact between the seed and the Pt${}^{*}$ layer, Ta is completely oxidized and above the pores between both layers there is pure Pt. The interdiffusion between Ta and Pt${}^{*}$ is restricted to the lower few 100 nm of the Pt${}^{*}$ film. There is no sharp transition between the Ta oxide and the Pt${}_{100-x}$Ta${}_{x}$ phase, but there is a fine grained transition zone. The composition of the Ta oxide is close to Ta${}_{2}$O${}_{5}$, which is the only well established equilibrium phase in the Ta-O phase system [31]. The density of Ta${}_{2}$O${}_{5}$ is about half that of Ta [32], which leads to a strongly increased layer thickness as is observed in the cross-section images.

In contrast to the other samples, the smooth sample surface with its facet like structure forms at lower temperatures (600 ${}^{\circ }$C) as can be seen in the SEM images in Figure 7c.

After the heat treatment at 800 ${}^{\circ }$C a two layer structure is visible (Figure 7d). The seed layer is continuously present and large grains exist in the Pt${}^{*}$ film. It has to be assumed that heating to higher temperatures than 600 ${}^{\circ }$C favors the complete oxidation of the Ta instead of the diffusion into the Pt. At the interface between both layers locally large pores have formed. No further changes are observed for the annealing at 1000 ${}^{\circ }$C (Figure 7e).

#### 3.2.4. Cr Seed Layer

**Figure 9:**
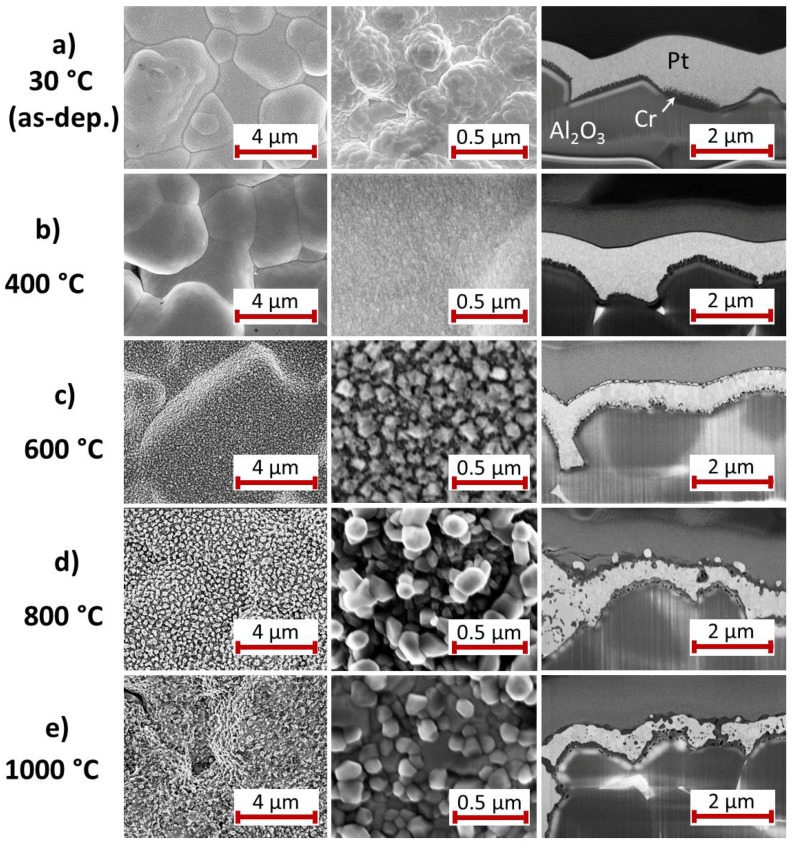
SEM images (InLens, 5 kV, 3 mm working distance) with two different magnifications of the surface of the Pt${}^{*}$ film on Cr seed layer (**a**) after deposition and after annealing for 2 h in air at (**b**) 400; (**c**) 600; (**d**) 800 and (**e**) 1000 ${}^{\circ }$C. On the right hand side a SEM image (InLens, 3 kV, 5 mm working distance) of the film cross-section is presented.

An overview of the surface morphology and cross-section images of the Pt${}^{*}$ film with Cr seed layer is given in Figure 9.

In contrast to the other seed layers there is no clear interface between the Cr and the Pt${}^{*}$ layer even at RT (Figure 9a). It appears that the Cr film is formed of fine columns. It has to be remarked that Cr dissolves as Cr(II) and Cr(III) ions in the ratio Cr(II):Cr(III) ≈ 7:1 by the sulfuric acid [33,34], which is part of the electrolyte used in the electrodeposition process. This reaction might lead to the observed grain structure in the Cr layer. A chemical analysis of the Pt${}^{*}$ film reveals that it contains no Cr so that the etched Cr atoms are not redeposited during the Pt${}^{*}$ deposition.

Heating the sample at 400 ${}^{\circ }$C leads to a smoothening of the surface. The cross-section image hardly shows a grain growth inside the Pt${}^{*}$ layer (Figure 9b); a fine grained structure is still visible.

The sample morphology strongly changes during the annealing at 600 ${}^{\circ }$C (Figure 9c). The surface images show large separated grains. The cross-section reveals some kind of three layer structure with about 100 nm sized grains in the middle Pt${}^{*}$ layer. At the surface there are brighter and darker grains and at the interface to the substrate a very inhomogeneous structure has formed with smaller grains.

**Figure 10:**
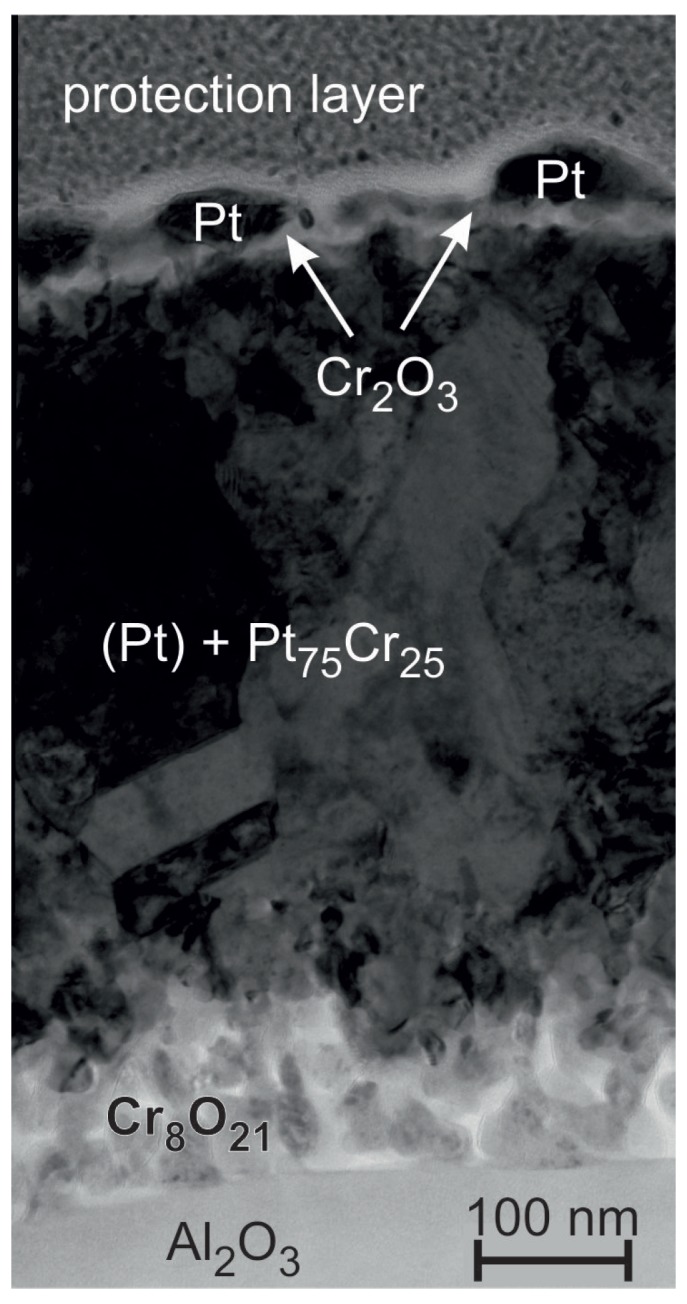
TEM image and EDX analysis of the Pt${}^{*}$ film on Cr seed layer after annealing at 600 ${}^{\circ }$C in air. The Cr-O phases are likely Cr${}_{2}$O${}_{3}$ at the upper part of the film and Cr${}_{8}$Cr${}_{21}$ at the interface to the substrate.

Furthermore, for this sample a TEM and EDX analysis was done to investigate details of the layered microstructure (Figure 10). On top of the substrate a Cr oxide layer has formed. The determination of the ratio between Cr and O with EDX is challenging since Cr has EDX peaks at the same position like O and there exist a lot of different Cr-O phases (see e.g., [35]). The calculated Cr:O ratio at the positions between substrate and the Pt${}^{*}$-Cr layer corresponds approximately to the Cr${}_{8}$O${}_{21}$ phase.

Cr was found across the whole thickness of the Pt${}^{*}$ layer. Most measured positions exhibit a composition between 22 and 28 at % of Cr, which fits to the Pt${}_{3}$Cr phase, which was also proven by the XRD measurements. According to the Pt-Cr phase diagram there is a solubility of more than 10 at % of Cr in Pt [36] (there are also phase diagrams which state a solubility of more than 50 at % Cr in Pt [37]). Locally a smaller Cr content was detected by the EDX measurements. However, we cannot distinguish if these are Pt grains with a smaller Cr content or if there is a superposition of a Pt matrix with Pt${}_{3}$Cr grains.

On top of the Pt${}_{3}$Cr film a thin Cr oxide layer is present, but with a lower O content (close to Cr${}_{2}$O${}_{3}$) as compared to the Cr oxide at the interface to the substrate (most likely Cr${}_{8}$O${}_{21}$). The phase Cr${}_{8}$O${}_{21}$ is an intermediate phase in the CrO${}_{3}$ decomposition process leading to the Cr${}_{2}$O${}_{3}$ final phase [38]. A possible explanation for the different Cr-O phases at the interface to the substrate and at the surface might be that the oxidation reaction is advanced at the surface with the direct contact to the ambient O as compared to the inner Cr-O below the Pt/Pt${}_{3}$Cr film.

The EDX measurements reveal that the individual grains, which appear bright in the cross-section images on top of the film are composed of pure Pt. In contrast to the body of the film, which is composed of a Pt-Cr phase, the Pt grains at the surface do not contain Cr.

After the heat treatment at 800 ${}^{\circ }$C the grains at the surface have further increased in size and thus decreased in number (Figure 9d). The contrast between the grains inside the Pt${}^{*}$ layer has reduced. In contrast to the 600 ${}^{\circ }$C sample a lot of dark features are present within the Pt${}^{*}$ film. The Pt${}^{*}$ layer is enclosed on both sides by a layer with a darker contrast, which corresponds most likely to the CrO${}_{x}$ layers. At the interface to the substrate pores have formed.

The bright grains at the sample surface that are visible in the cross-sections are strongly reduced in number and size if the annealing temperature is increased to 1000 ${}^{\circ }$C (Figure 9e). Obvious is the formation of pores at the interface to the substrate. The thin darker layers on top of the sample and at the interface to the substrate are still present. Locally there are connections between these two layers through the Pt${}^{*}$ film.

**Figure 11:**
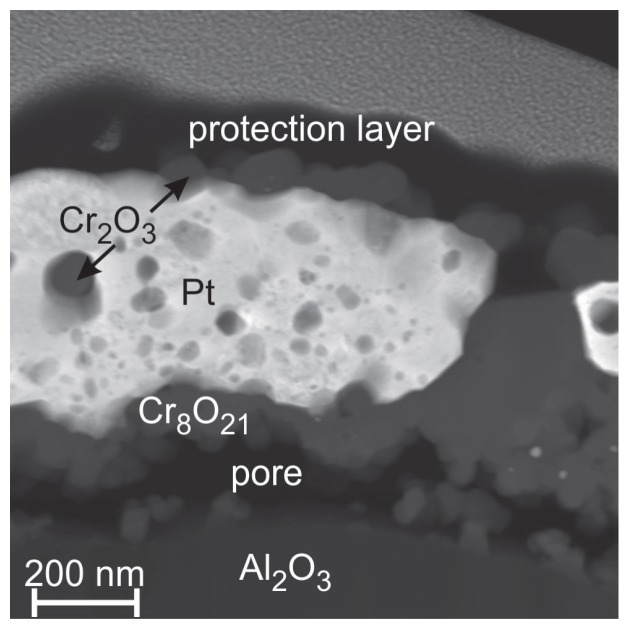
STEM image (chemical contrast) of the Pt${}^{*}$ film on Cr seed layer after annealing at 1000 ${}^{\circ }$C in air.

It is known that at higher temperatures (between 600 and 900 ${}^{\circ }$C) Cr${}_{2}$O${}_{3}$ forms with O${}_{2}$ gaseous CrO${}_{3}$ [39]. This behavior leads to the increasing number of pores if the sample is heated at increasingly higher temperatures. Figure 11 shows a STEM image (chemical contrast) of the sample annealed at 1000 ${}^{\circ }$C. The EDX measurements reveal a demixing of the Pt and Cr. A pure Pt matrix is left in which spherical Cr oxide (likely Cr${}_{2}$O${}_{3}$) grains are embedded. The Cr oxide layer on top is not continuous. As for the 600 ${}^{\circ }$C sample there is the compositional difference between the Cr oxide on top and that at the interface to the substrate.

In contrast to all other films, a facet like surface structure is not formed at all for this system.

#### 3.2.5. Pt Seed Layer

**Figure 12:**
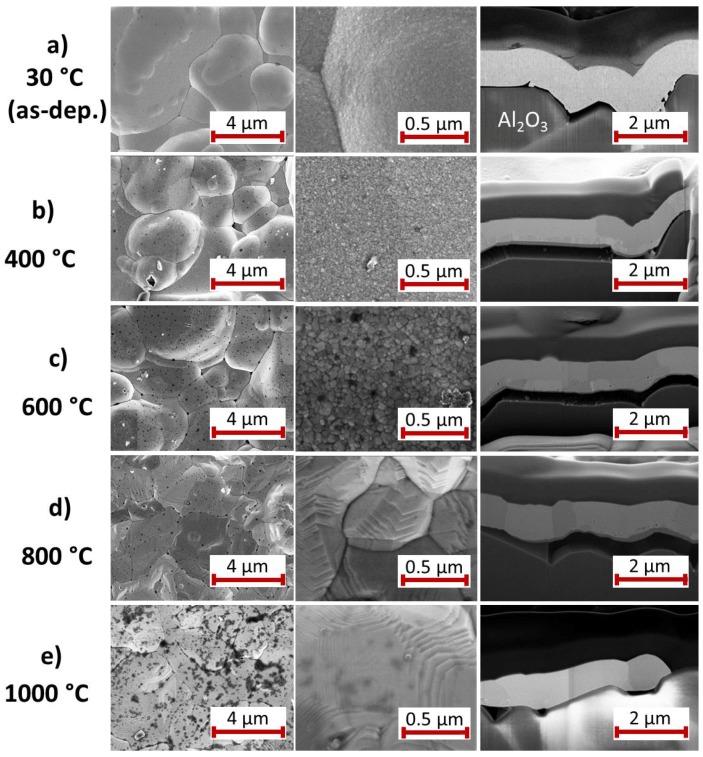
SEM images (InLens, 5 kV, 3 mm working distance) with two different magnifications of the surface of the Pt${}^{*}$ film on Pt seed layer (**a**) after deposition and after annealing for 2 h in air at (**b**) 400; (**c**) 600; (**d**) 800 and (**e**) 1000 ${}^{\circ }$C. On the right hand side an SEM image (InLens, 3 kV, 5 mm working distance) of the film cross-section is presented.

Figure 12 summarizes the surface and cross-section images of the Pt${}^{*}$ film on a Pt seed layer.

In contrast to all the other bilayers, already at RT there is hardly any contact between the Pt film stack and the substrate (Figure 12a). A connection between the film and the substrate exists only at few distinct points, otherwise large gaps are visible because of partial delamination. Images of the cross-section with higher magnification reveal that there are many small pores at the interface between the Pt seed and the Pt${}^{*}$ layer. In both layers small grains (few tens of nm) are visible.

The grains at the sample surface have slightly grown after annealing at 400 ${}^{\circ }$C (Figure 12b). An interface between both Pt films is still visible. While grains in the Pt${}^{*}$ layer are extended over several *μ*m, those in the Pt film are significantly smaller (in the 100 nm range). The gaps between the Pt film and the substrate have become larger due to intrinsic stresses during the heat treatment. Locally there is a distance of several 100 nm.

The heat treatment at 600 ${}^{\circ }$C leads to a further growth of the grains at the sample surface and in the Pt seed layer (Figure 12c). The thickness of the former Pt seed layer increases at the expense of the thickness of the Pt${}^{*}$ layer. Still visible are pores at the grain boundaries of the Pt seed layer and between the Pt seed and the Pt${}^{*}$ layer.

After annealing at 800 ${}^{\circ }$C the facet-like sample surface has developed (Figure 12d). Most of the grains in the Pt film are now extended over the whole film thickness, only few single smaller grains are present near to the interface to the substrate. With the coalescence of the seed and Pt${}^{*}$ layer most of the pores disappear.

Finally, the heat treatment at 1000 ${}^{\circ }$C leads to a Pt film with large grains. No bilayer structure is visible anymore (Figure 12e). Clear facets are present at the sample surface.

### 3.3. Electrical Properties

**Figure 13:**
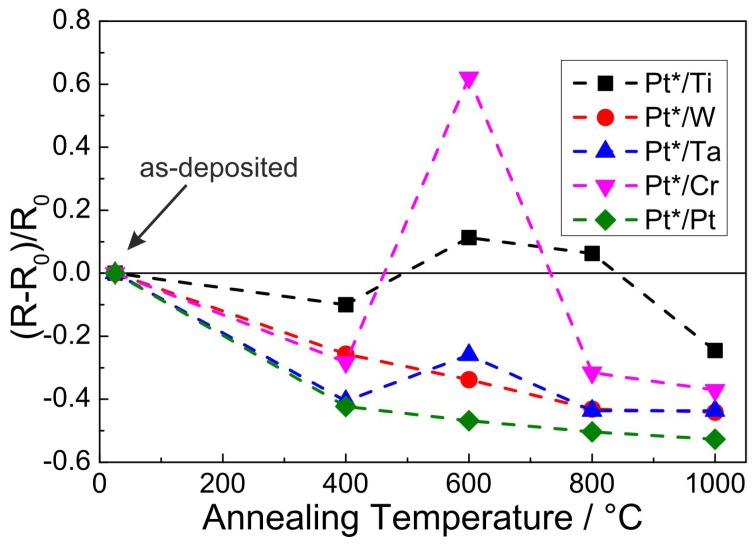
Relative electrical resistance of the Pt${}^{*}$-seed bilayers with respect to the individual resistance values $R_{0}$ measured for the as-deposited samples after the annealing steps.

Since the films were investigated to evaluate their applicability as a material for the preparation of antennas on Al${}_{2}$O${}_{3}$ ceramics, besides the film morphology the electrical resistance is crucial. Figure 13 summarizes the values of the electrical resistance of the films with the different seed layers in the as-prepared state and after the different annealing steps. The values are all measured after the heat treatment at RT. Since all films possess a different “mean” film thickness (due to the extreme roughness of the substrate the declaration of a concrete film thickness is not possible) the absolute electrical resistance is not a practical value to describe and compare the different samples. Therefore, the change of the resistance with temperature is presented in such a way that the individual values *R* are normalized to the value measured for the as-prepared state $R_{0}$. Each measurement point in Figure 13 represents the mean value of eight individual measurements with alternating direction of the electrical current. The standard deviation of the measurements for each point is less than 0.1% and with this smaller than the respective symbols in the plot.

The first annealing step at 400 ${}^{\circ }$C leads to a decrease of the electrical resistance for all samples. The lowest change is observed for the Ti seed layer (−10%) followed by the W and Cr seed layers (≈−25%). The strongest reduction occurs for the Ta and Pt seed layer (≈−40%).

Increasing the annealing temperature to 600 ${}^{\circ }$C leads to a slight further reduction of the electrical resistance for the W and Pt seed layer (≈−35% and ≈−45%, respectively). For the Ti seed layer, the electrical resistance slightly increases above the RT value (≈+10%). Furthermore, for the Ta seed layer there is a slight increase compared to the value measured after the heat treatment at 400 ${}^{\circ }$C. In contrast to all other samples, the film with the Cr seed layer shows a strong increase to +62% as compared to the RT value.

After the heat treatment at 800 ${}^{\circ }$C the electrical resistance for the Cr sample strongly decreases to a value comparable to that measured after annealing at 400 ${}^{\circ }$C (−32%). For all other samples there is a slight reduction as compared to the 600 ${}^{\circ }$C value.

The heat treatment at 1000 ${}^{\circ }$C leads to a further decrease of the electrical resistance for all samples. In summary we find a continuous decrease of the electrical resistance for the W and Pt seed layer. The other samples show an increase after annealing at 600 ${}^{\circ }$C followed by a decrease at higher temperatures. The lowest relative value after annealing at 1000 ${}^{\circ }$C is reached by the Pt${}^{*}$-Pt sample (−53%), followed by the Pt${}^{*}$-W and Pt${}^{*}$-Ta sample (−44%) and the Pt${}^{*}$-Cr sample (−37%). The highest relative value is reached for the Pt${}^{*}$-Ti sample with a reduction compared to the as-deposited state of −25%.

## 4. Discussion

For the interpretation of the measurements one has to be aware that the different temperature steps were independent, i.e., for each annealing experiment a “new” sample in the as-deposited state was used. Therefore, effects that appear at a certain temperature—e.g., 600 ${}^{\circ }$C seems to be a critical temperature step especially for interdiffusion effects—might not take place if the sample is directly heated (10 K/min) to the higher temperatures. Other competing effects like oxidation of the seed layer material may then be favored.

**Figure 14:**
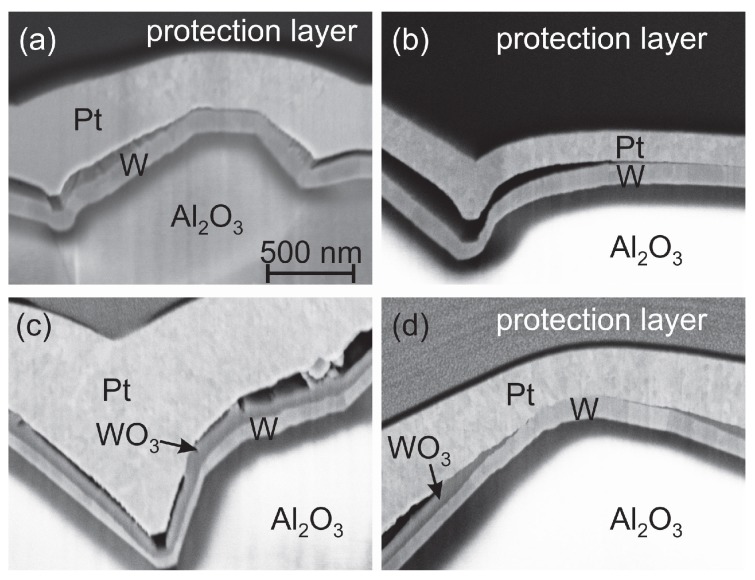
Cross-section images of a Pt${}^{*}$/W/Al${}_{2}$O${}_{3}$ sample in (**a**) the as-prepared state and after annealing (**b**) in forming gas and (**c**) in air at 400 ${}^{\circ }$C for 2 h; (**d**) sample annealed in air showing a position where no WO${}_{3}$ has formed since there is no gap between the Pt${}^{*}$ and the barrier layer.

We found a complete oxidation of the Ti, W, Ta and Cr seed layer. Either the seed layer oxide still exists as an individual layer (e.g., in the case of WO${}_{3}$ and to a large extend for Ta${}_{2}$O${}_{5}$) or the oxide grains are distributed between (TiO${}_{x}$) or below, above and within (CrO${}_{x}$) the Pt${}^{*}$ grains. The oxidation of the seed layer starts from the side of the Pt${}^{*}$ film; however the Pt film itself hardly contains any O. To elucidate the origin of the O another experiment was performed. The sample with W barrier layer was chosen since it shows a clear bilayer structure consisting of W and WO${}_{3}$ after annealing at 400 ${}^{\circ }$C. In contrast to the first experiments with annealing under air conditions this time we chose a forming gas atmosphere. Figure 14 shows a cross-section image of the as-deposited Pt${}^{*}$/W/Al${}_{2}$O${}_{3}$ sample (Figure 14a) together with the image of the same sample (lower Pt${}^{*}$ thickness because of different sample position) annealed in forming gas at 400 ${}^{\circ }$C (Figure 14b) and the reference image after the annealing under air conditions (Figure 14c). The results clearly show that the annealing in forming gas does not lead to an oxidation of the W seed layer. The W grains are still visible as observed in the as deposited state. Therefore we conclude that during the annealing small cracks are formed in the Pt${}^{*}$ layer so that the gaps between the Pt${}^{*}$ layer and the W barrier are filled with air leading to the oxidation of the W layer. Obvious is the constant thickness of the WO${}_{3}$ and W layers. However, we find positions without a delamination gap between the Pt${}^{*}$ and the W film where no W oxide has formed (Figure 14d). This leads to the assumption that the Pt${}^{*}$ layer itself is dense and only local defects within the film act as O diffusion paths.

Nevertheless, all seed layers lead to a stable Pt film on the Al${}_{2}$O${}_{3}$. Although the Pt layer is locally delaminated from the substrate, the film does not peel off.

### 4.1. Ti Seed Layer

For the samples with the Ti seed layer the XRD measurements show a strong phase formation and grain growth after annealing at 400 ${}^{\circ }$C. The grain growth from RT to 400 ${}^{\circ }$C leads to the observed reduction in the electrical resistance. The cross-section and STEM image of the sample annealed at 600 ${}^{\circ }$C (Figure 3c and Figure 4a) reveal a strong grain boundary contrast in the Pt${}^{*}$ film caused by Ti and Ti oxide diffusion along these structures, which is also the origin of the increase of the electrical resistance for this annealing temperature. After the heat treatment at 800 ${}^{\circ }$C hardly any grain boundary contrast is found, however few pores and TiO${}_{x}$ are left between some grains. All together this leads to the slight reduction in electrical resistance. The ongoing grain growth at higher temperatures is also visible in the further increased Pt XRD peaks and the lower electrical resistance.

### 4.2. W Seed Layer

In contrast to all other samples there is no reaction between the W seed and the Pt${}^{*}$ layer, which explains a similar development of the electrical resistance as compared to the Pt${}^{*}$/Pt bilayer. The oxidation of the W layer between 400 and 600 ${}^{\circ }$C leads to a peeling of the Pt${}^{*}$ film but has no obvious influence on the grain growth of Pt${}^{*}$ with increasing temperature. The WO${}_{3}$ formation starts from the Pt side of the samples and not from the substrate interface. There is a continuous reduction of the electrical resistance in the whole temperature range. XRD measurements show WO${}_{3}$ reflexes for the 600 and 800 ${}^{\circ }$C annealing step, but not for the 1000 ${}^{\circ }$C sample. As noticed before, WO${}_{3}$ starts to sublime substantially above 900 ${}^{\circ }$C, which explains the large pores that are visible in the respective cross-section image and the disappearance of the XRD WO${}_{3}$ reflexes.

### 4.3. Ta Seed Layer

The XRD measurements show a strong grain growth at 400 ${}^{\circ }$C but lower intensities at the higher temperatures. At 600 ${}^{\circ }$C the cross-section image as well as the TEM investigation reveal a strong reaction between the Ta barrier and Pt${}^{*}$ layer, which explains the reduction in the Pt XRD peaks and the increased electrical resistance. The phase diagram Pt-Ta shows a high solubility of Ta in Pt of about 15 at % at 600 ${}^{\circ }$C and several intermetallic phases.

The observed grain structures at 600 ${}^{\circ }$C are not visible for the films annealed at higher temperatures. One reason might be that the high temperatures favor the oxidation instead of the interdiffusion of Ta and Pt. We assume that the Ta oxide formation occurs faster than the intermetallic phase formation and that at high temperatures the Ta layer has oxidized completely. The grain growth and the demixing then lead to the decreased electrical resistance.

### 4.4. Cr Seed Layer

Of all investigated seed layers we found the strongest reaction between the Cr seed and the Pt${}^{*}$ layer. Different at RT, compared to the other films, is the rough interface between the Cr and Pt${}^{*}$ layer. The phase formation and change in resistance upon annealing at 400 ${}^{\circ }$C is comparable to the other samples. However, after annealing at 600 ${}^{\circ }$C there are strong differences compared to the other samples: XRD shows a two phase state with only a small Pt peak and a Pt${}_{3}$Cr peak beside it and the cross-section image reveals a small grained inhomogeneous structure. This explains the strong increase in electrical resistance. Cr diffuses through the whole Pt layer, which leads to the observed formation of the Cr oxide layer, not only at the bottom, but also on top of the Pt/PtCr${}_{x}$ film.

Obviously, the annealing temperature of 400 ${}^{\circ }$C is not sufficient for the Pt${}_{3}$Cr phase formation. On the other hand, at higher annealing temperatures of 800 and 1000 ${}^{\circ }$C, this phase is also not detected, although it is stable at higher temperatures according to the phase diagram [36]. For these temperatures, it has to be assumed that the oxidation kinetics of Cr dominates the Pt${}_{3}$Cr phase formation. The slightly higher electrical resistance of the sample after annealing at 1000 ${}^{\circ }$C as compared to the others (besides the sample with Ti) can be explained by the numerous CrO${}_{x}$ grains, which are distributed in the Pt layer and act as scattering centers.

### 4.5. Pt Seed Layer

In case of the Pt seed layer, the XRD measurements show, like for the other films, a strong grain growth and phase formation for the 400 ${}^{\circ }$C sample. Of course, in contrast to the other materials, interdiffusion or intermetallic phase formation do not play a role. As stated above, the development of the electrical resistance of the Pt${}^{*}$/Pt film is quite similar to that of the Pt${}^{*}$/W system. In the case of W, due to the formation of W oxide there is also no reaction with the Pt${}^{*}$ layer and there is hardly any contact between the Pt and the WO${}_{3}$ film, so that the development of the Pt${}^{*}$ grains takes place comparable to the Pt${}^{*}$/Pt sample.

### 4.6. Evaluation of the Adhesion

**Figure 15:**
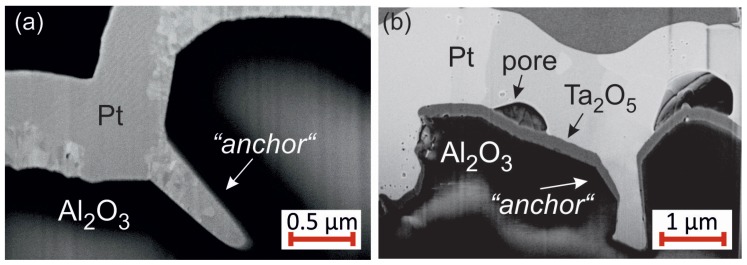
Example of anchor structures, which fix the Pt${}^{*}$ film to the Al${}_{2}$O${}_{3}$ substrate. (**a**) Pt seed layer, sample annealed at 400 ${}^{\circ }$C in air and (**b**) Ta seed layer, sample annealed at 800 ${}^{\circ }$C in air.

The FIB cuts of the Pt${}^{*}$ films with Pt seed layer show large pores between the substrate and the film. However, this film like all the others does not delaminate from the substrate and stays attached to it. Due to the high roughness of the substrate, at some positions “anchor-like” structures form as can be seen in the exemplary images in Figure 15 for samples with Pt (Figure 15a) or Ta (Figure 15b) seed layer. These “anchors” keep the film stuck to the substrate.

## 5. Conclusions

The investigations show several challenges for the application of seed layers for electrodeposited Pt films. On the one hand, there are interdiffusion effects; on the other hand, an oxidation of the applied seed materials takes place. Considering phase formation and electrical resistance, the Pt${}^{*}$/Pt system shows the best properties among the investigated materials. For all other systems, a complete oxidation of the seed layer is observed after the 2-h annealing process. If the oxide is distributed along the Pt grain boundaries (TiO${}_{x}$) or in addition, also randomly within the Pt grains (CrO${}_{x}$), the electrical properties are reduced. However, all investigated systems lead to a Pt film that is attached to the substrate and does not peel off.

Although cross-section images show only local contact between the Pt${}^{*}$/Pt system and the substrate, the film does not lift off, which we assume to be caused by the anchor-like structures.

As the films shall be used in SAW sensor technology working at high temperatures, it is essential that during the application, no phase transitions or oxidation effects take place. The authors especially notice these effects for the 600 ${}^{\circ }$C samples. From our experiments, we conclude that prior to the application, the sample needs to be annealed at high temperatures to achieve a stable state.

## Figures and Tables

**Table 1:** Deposition rates of the various seed layer materials.

Material	Ti	Cr	W	Ta	Pt
Deposition rate in nm/s	0.27	0.3	0.18	0.49	0.1

## References

[1] Thiele J.A., da Cunha M.P. (2005). Platinum and palladium high-temperature transducers on langasite. IEEE Trans. Ultrason. Ferroelectr..

[2] Moulzolf S.C., Frankel D.J., da Cunha P.M., Lad R.J. (2014). High temperature stability of electrically conductive Pt-Rh/ZrO_2_ and Pt-Rh/HfO_2_ nanocomposite thin film electrodes. Microsyst. Technol..

[3] Lin C.M., Yen T.T., Felmetsger V.V., Hopcroft M.A., Kuypers J.H., Pisano A.P. (2010). Thermally compensated aluminum nitride Lamb wave resonators for high temperature applications. Appl. Phys. Lett..

[4] Ciftyurek E., Sabolsky K., Sabolsky E.M. (2013). Platinum thin film electrodes for high-temperature chemical sensor applications. Sens. Actuators B Chem..

[5] Battistel D., Battaglin G., Daniele S. (2014). Platinum/alumina thin films prepared by r.f. magnetron sputtering as platforms in voltammetric sensing. Sens. Actuators B Chem..

[6] Weng Y.C., Rick J.F., Chou T.C. (2004). A sputtered thin film of nanostructured Ni/Pt/Ti on Al_2_O_3_ substrate for ethanol sensing. Biosens. Bioelectron..

[7] Plessky V.P., Reindl L.M. (2010). Review on SAW RFID Tags. IEEE Trans. Ultrason. Ferroelectr..

[8] Kang A.L., Zhang C.R., Ji X.J., Han T., Li R.S., Li X.W. (2013). SAW-RFID enabled temperature sensor. Sens. Actuators A Phys..

[9] Binder A., Bruckner G., Schobernig N., Schmitt D. (2013). Wireless Surface Acoustic Wave Pressure and Temperature Sensor With Unique Identification Based on LiNbO_3_. IEEE Sens. J..

[10] Saldanha N., Malocha D.C. (2012). Pseudo-Orthogonal Frequency Coded Wireless SAW RFID Temperature Sensor Tags. IEEE Trans. Ultrason. Ferroelectr..

[11] Fiaccabrino G.C., Koudelka-Hep M. (1998). Thin-film microfabrication of electrochemical transducers. Electroanalysis.

[12] Khanna V.K. (2011). Adhesion-delamination phenomena at the surfaces and interfaces in microelectronics and MEMS structures and packaged devices. J. Phys. D Appl. Phys..

[13] Cordill M.J., Moody N.R., Bahr D.F. (2004). Quantifying improvements in adhesion of platinum films on brittle substrates. J. Mater. Res..

[14] Bernhardt G., Silvestre C., le Cursi N., Moulzolf S.C., Frankel D.J., Lad R.J. (2001). Performance of Zr and Ti adhesion layers for bonding of platinum metallization to sapphire substrates. Sens. Actuators B Chem..

[15] Sreenivas K., Reaney I., Maeder T., Setter N., Jagadish C., Elliman R.G. (1994). Investigation of Pt/Ti Bilayer Metallization on Silicon for Ferroelectric Thin-Film Integration. J. Appl. Phys..

[16] Tiggelaar R.M., Sanders R.G.R., Groenland A.W., Gardeniers J.G.E. (2009). Stability of thin platinum films implemented in high-temperature microdevices. Sens. Actuators A Phys..

[17] Derniaux E., Kayser P., Gageant C., Sanchez C., Boivin D. (2006). Effects of TiO*_x_* physical vapor deposition parameters on the preferred orientation and adhesion of Pt films on gamma-Al_2_O_3_. J. Vac. Sci. Technol. A.

[18] Park K.H., Kim C.Y., Jeong Y.W., Kwon H.J., Kim K.Y., Lee J.S., Kim S.T. (1995). Microstructures and Interdiffusions of Pt/Ti Electrodes with Respect to Annealing in the Oxygen Ambient. J. Mater. Res..

[19] Ehrlich A., Weiss W., Hoyer W., Gessner T. (1997). Microstructural changes of Pt/Ti bilayer during annealing in different atmospheres - an XRD study. Thin Solid Films.

[20] Maeder T., Sagalowicz L., Muralt P. (1998). Stabilized platinum electrodes for ferroelectric film deposition using Ti, Ta and Zr adhesion layers. Jpn. J. Appl. Phys..

[21] Tisone T.C., Drobek J. (1972). Diffusion in thin-film Ti-Au, Ti-Pd, and Ti-Pt couples. J. Vac. Sci. Technol..

[22] Ciftyurek E., McMillen C.D., Sabolsky K., Sabolsky E.M. (2015). Platinum-zirconium composite thin film electrodes for high-temperature micro-chemical sensor applications. Sens. Actuators B Chem..

[23] Shelton C.T., Kotula P.G., Brennecka G.L., Lam P.G., Meyer K.E., Maria J.P., Gibbons B.J., Ihlefeld J.F. (2012). Chemically Homogeneous Complex Oxide Thin Films Via Improved Substrate Metallization. Adv. Funct. Mater..

[24] Pauw L.J.v.d. (1958). A method of measuring the resistivity and Hall coefficient on lamellae of arbitrary shape. Philips Res. Rep..

[25] Li M., Han W., Li C. (2008). Thermodynamic assessment of the Pt-Ti system. J. Alloy. Compd..

[26] Biggs T., Cornish L.A., Witcomb M.J., Cortie M.B. (2004). Revised phase diagram for the Pt-Ti system from 30 to 60 at.% platinum. J. Alloy. Compd..

[27] Wahlbeck P.G., Gilles P.W. (1966). Reinvestigation of the phase diagram for the system titanium-oxygen. J. Am. Ceram. Soc..

[28] Slade P.G. (2013). Electrical Contacts: Principles and Applications.

[29] Lassner E., Schubert W.-D. (2012). Tungsten: Properties, Chemistry, Technology of the Element, Alloys, and Chemical Compounds.

[30] Waterstrat R.M. (1981). Analysis of selected alloys in the system Cr-Pd, Cr-Ru, V-Pd and Ta-Pt. J. Less-Common Met..

[31] Garg S.P., Krishnamurthy N., Awasthi A., Venkatraman M. (1996). The O-Ta (Oxygen-Tantalum) system. J. Phase Equilibr..

[32] Reisman A., Holtzberg F., Berkenblit M., Berry M. (1956). Reactions of the Group VB Pentoxides with Alkali Oxides and Carbonates. III. Thermal and X-ray Phase Diagrams of the System K_2_O or K_2_CO_3_ with Ta_2_O_5_. J. Am. Chem. Soc..

[33] Drazic D.M., Popic J.P. (2002). Dissolution of chromium in sulfuric acid. J. Serb. Chem. Soc..

[34] Popic J.P., Drazic D.M. (2004). Electrochemistry of active chromium—Part ii. Three hydrogen evolution reactions on chromium in sulfuric acid. Electrochim. Acta.

[35] Norby P., Christensen A.N., Fjellvag H., Nielsen M. (1991). The crystal structure of Cr_8_O_21_ determined from powder diffraction data: Thermal transformation and magnetic properties of a chromium-chromate- tetrachromate. J. Solid State Chem..

[36] Baglin J.E.E., D’Heurle F.M., Zirinsky S. (1978). Interaction between Cr and Pt films: New Cr-Pt Phases. J. Electrochem. Soc..

[37] Aldinger F. (1995). Thermodynamic models and data for pure elements and other endmembers of solutions. Group 6: Periodic system effects. CALPHAD: Comput. Coupling Phase Diagrams Thermochem..

[38] Norby P., Fjellvag H. (2003). Thermal Decomposition of Chromium(VI) Oxide; High Resolution In-Situ Powder Diffraction Studies of Mixed Valence Chromium Oxides.

[39] Stanislowski M., Wessel E., Hilpert K., Markus T., Singheiser L. (2007). Chromium Vaporization from High-Temperature Alloys: I. Chromia-Forming Steels and the Influence of Outer Oxide Layers. J. Electrochem. Soc..

